# Identification of Oil Palm’s Consistently Upregulated Genes during Early Infections of *Ganoderma boninense* via RNA-Seq Technology and Real-Time Quantitative PCR

**DOI:** 10.3390/plants10102026

**Published:** 2021-09-27

**Authors:** Liyana Mohd Zuhar, Ahmad Zairun Madihah, Siti Aqlima Ahmad, Zamri Zainal, Abu Seman Idris, Noor Azmi Shaharuddin

**Affiliations:** 1 Department of Biochemistry, Faculty of Biotechnology and Biomolecular Sciences, Universiti Putra Malaysia UPM, Serdang 43400, Selangor, Malaysia; mizleeyana@gmail.com (L.M.Z.); aqlima@upm.edu.my (S.A.A.); 2 Malaysian Palm Oil Board, 6 Persiaran Institusi, Bandar Baru Bangi, Kajang 43000, Selangor, Malaysia; madihah@mpob.gov.my (A.Z.M.); idris@mpob.gov.my (A.S.I.); 3 Department of Biological Sciences and Biotechnology, Faculty of Science and Technology, Universiti Kebangsaan Malaysia UKM, Bangi 43600, Selangor, Malaysia; zz@ukm.edu.my; 4 Institute of Plantation Studies, Universiti Putra Malaysia UPM, Serdang 43400, Selangor, Malaysia

**Keywords:** oil palm, transcriptomic library construction, transcript expression profile, *Elaeis guineensis* Jacq, *Ganoderma boninense*

## Abstract

Basal stem rot (BSR) disease caused by pathogenic fungus *Ganoderma boninense* is a significant concern in the oil palm industry. *G. boninense* infection in oil palm induces defense-related genes. To understand oil palm defense mechanisms in response to fungal invasion, we analyzed differentially expressed genes (DEGs) derived from RNA-sequencing (RNA-seq) transcriptomic libraries of oil palm roots infected with *G. boninense.* A total of 126 DEGs were detected from the transcriptomic libraries of *G. boninense*-infected root tissues at different infection stages. Functional annotation via pathway enrichment analyses revealed that the DEGs were involved in the defense response against the pathogen. The expression of the selected DEGs was further confirmed using real-time quantitative PCR (qPCR) on independent oil palm seedlings and mature palm samples. Seven putative defense-related DEGs consistently showed upregulation in seedlings and mature plants during *G. boninense* infection. These seven genes might potentially be developed as biomarkers for the early detection of BSR in oil palm.

## 1. Introduction

Oil palm (*Elaeis guineensis* Jacq.) is one of Malaysia’s most economically significant plantation crops, with approximately 5.90 million hectares of reported cultivation in 2019 [1,2]. The highest oil-yielding crop contributes 33.3% of the world’s palm oil production, with export revenue earnings of more than USD 18 billion [3,4]. However, Malaysian oil palm cultivation suffers from a devastating disease known as basal stem rot (BSR), caused by the fungus *Ganoderma boninense*. BSR is a fatal disease in the oil palm industry in Malaysia. It causes the internal trunk tissue to rot, thus reducing the number of standing palms. Therefore, the threat of BSR disease towards the oil palm industry warrants extensive effort in finding a solution to eradicate this disease permanently.

The BSR-affected area in Malaysia totals approximately 151,208 ha, causing a loss of RM 1.3 billion. The regions affected by BSR incidence were estimated to increase to 400,000 ha by 2020 [5]. Several BSR control measures, such as sanitation [6], trenching systems [7], chemical control (i.e., hexaconazole and the combination of the carboxin and quintozene fungicides) and biological control agents (*Trichoderma* spp.) [8] have continued to give inconsistent results as half of the internal palm tissues were already rotten during the first appearance of BSR symptoms [9].

Oil palms from different geographical origins have been reported to have different levels of susceptibility and resistance towards *G. boninense* infection [10,11,12]. The most commonly planted palms in Malaysia are the susceptible variety, *Tenera* [13]. Oil palm planting materials have been produced sexually through *Dura x Pisifera* (DxP), which results in the *Tenera* offspring. The DxP materials (*Tenera*) are significantly superior to their parental palms. The higher yield production of *Tenera* oil, as compared to the *Dura* and *Pisifera*, has given this palm high commercial value [14]. In Malaysia, the *Tenera* variety can yield around 4 to 5 tonnes of palm oil and around 1 tonne of palm kernels per hectare per year, respectively. Many defense-related genes have been identified and characterized from this variety, including several genes that contribute to the virulence of *G. boninense*, such as chitinases, isoflavone reductase, glucanases, stearoyl-acyl carrier protein desaturase (SAD) and metallothionein-like protein [15,16,17,18,19]. However, information on genes in response to *G. boninense* infection is limited [20] and inadequate to generate a complete picture of oil palm’s defense response towards *G. boninense* infection. 

RNA-sequencing (RNA-seq), a sequencing technology capable of reconstructing a large number of full-length RNA transcripts within a single reaction, has been proven to successfully characterize plant defense mechanisms [21,22,23,24,25], including in *G. boninense*- infected oil palm [13,20,26]. It was reported that different sets of oil palm defense-related genes were triggered at various stages of BSR infection. Therefore, RNA-seq analysis has the potential to discover new genes involved in the molecular interaction between oil palm and *G. boninense* during different stages of fungal infection.

In the present study, comprehensive work was planned to analyze and identify differentially expressed genes (DEGs) from RNA-seq transcriptomic libraries of oil palm roots infected with *G. boninense*. Some selected DEGs’ expression profiles were further confirmed using quantitative polymerase chain reaction (qPCR) on BSR-infected oil palm samples.

## 2. Results

### 2.1. Transcriptomic Analysis via RNA-Seq

To identify potential genes that are specifically involved in the defense mechanisms of oil palm roots towards *G. boninense* infection, we harvested oil palm roots of *Tenera* that were artificially infected with the fungus *G. boninense* at two different times of infection, labeled as early (scale 1) and late (scale 3) infection categories. The classification of the fungus infection stage was made based on the BSR severity score on a scale of 0–4, with 0 indicating a healthy-looking palm without any fungal mycelium appearance and 4 regarded as a dead palm. For the early infection category, the samples were harvested based on the observation of lesions and mycelia on the root surface at 3 to 12 weeks post-inoculation without any foliar symptoms observed. As for the late infection category, the samples were harvested based on the observation of disease symptoms such as yellowing of leaves and the formation of fruiting bodies on the stem. The RNA of the root tissues from three biological replicates were extracted and then pooled for each category in each oil palm treatment to reduce the potential variability present among individual samples and increase the capacity to detect differential gene expression between each category of sample treatment [13,27,28,29]. 

Before the RNA-seq, the integrity of the RNA samples was determined, and only RNA with high RIN values (5–8) were subjected to sequencing. From the RNA-seq analysis, four (4) transcriptomics libraries were constructed, namely root early infection (REI), root late infection (RLI), root early control (REC) and root late control (RLC). We acquired 15 million, 19 million, 16 million and 24 million sequence reads from REI, RLI, REC and RLC, respectively. A summary of the data processing analysis is shown in Table 1.

### 2.2. Identification of DEGs

The DEGs were identified after the reads were mapped to the oil palm reference transcriptome sequence to compare the transcripts expressed in oil palm root samples during the colonization of *G. boninense*. Both datasets were compared between transcripts from early infection (REI/REC) and late infection (RLI/RLC) via Cuffdiff and Fragments Per Kilobase of transcript per Million reads mapped (FPKM) value. Based on the result, a total of 126 DEGs were obtained, as shown in Table 2. A clear inclining trend in the values of DEG expression (total DEGs, upregulated transcript and downregulated transcript) was observed from the early to the late infections. At the early infection stage, only 21 DEGs were detected (12 upregulated and 9 downregulated transcripts). The numbers of DEGs increased to 103 (69 upregulated and 34 downregulated transcripts) in the late infection stage. 

### 2.3. Functional Annotation

The Gene Ontology (GO) program was used to annotate and map the identified DEGs to specific biological pathways and gene metabolic pathways. At the early infection stage (REI/REC library), the highest GO term categorization was assigned to biological processes, with 48.2% DEGs corresponding to nitrate, somatic embryogenesis, chitin catabolic process and respiratory burst. Meanwhile, 21% DEGs were mapped to cellular components, with cytoplasmic, membrane-bonded vesicle, plant-type cell wall and plasma membrane as the most common of the group. The remaining 30.9% DEGs were grouped into molecular functions, with involvement in iron-binding, chitinase activity and chitin-binding as the group’s primary function. 

**Figure 1:**
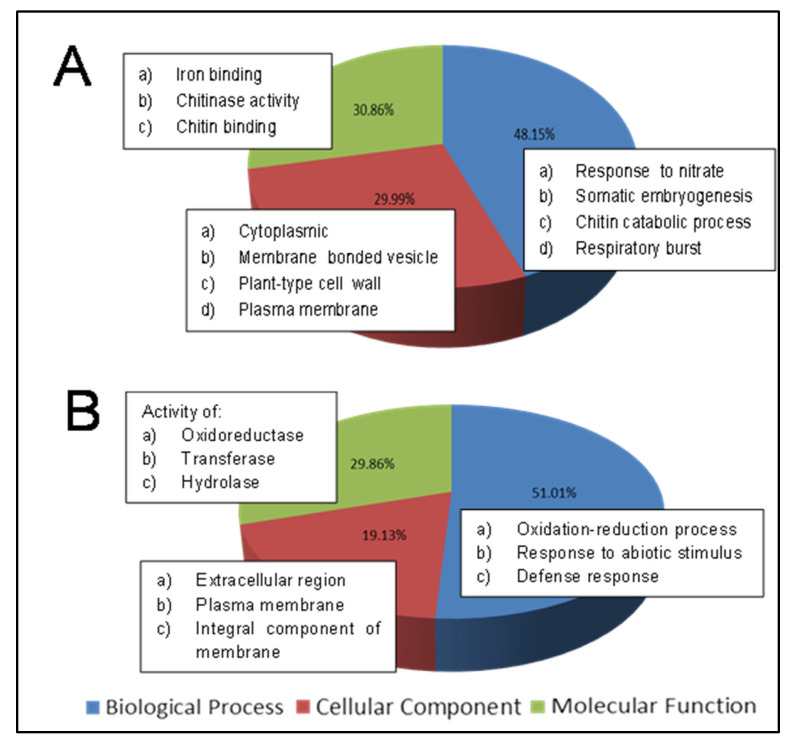
Gene Ontology (GO) clustering analysis for both root treatments. GO characterized the DEGs into biological processes, molecular functions and cellular components. (**A**) Root early infection genes (REI/REC) and (**B**) root late infection genes (RLI/RLC).

Meanwhile, at the late infection stage (RLI/RLC library), the highest GO term categorization was assigned to biological processes, containing 51% DEGs, with the oxidation–reduction process, response to abiotic stimulus and defense response as the most prominent biological processes of the group. Moreover, 19.1% DEGs were assigned to cellular components, with the extracellular region, plasma membrane and integral component of the membrane as the most frequent of the group. Lastly, 24.9% DEGs were assigned to molecular functions, with involvement in oxidoreductase activity, transferase activity and hydrolase activity as the major functions of the group. The GO categorizations for both REI/REC and RLI/RLC libraries are depicted in Figure 1.

The relationships among the DEGs and their functions were further visualized via Kyoto Encyclopedia of Genes and Genomes (KEGG) pathway visualization. The KEGG pathway enrichment analysis of REI/REC libraries showed that the DEGs involved in three pathway classes with the pathway class of nitrogen metabolisms were the most represented. Meanwhile, the enrichment analysis showed that the DEGs from RLI/RLC sample libraries involved in 29 pathway classes with the majority of the DEGs were represented in the pathway class of the metabolism of xenobiotics by cytochrome P450. 

### 2.4. Gene Expression Profiling of Selected DEGs via qRT-PCR

**Figure 2:**
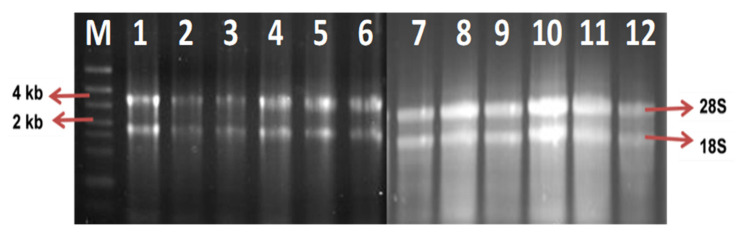
Total RNA of oil palm root tissues from oil palm seedlings (lanes 1–5) and mature palms (lanes 7–12) on 1.5% (*w*/*v*) agarose gel electrophoresis showed distinct 28S and 18S rRNAs bands. M: GeneRuler RNA Ladder High Range (Fermentas, Foster City, CA, USA).

In this study, 10 DEGs with upregulation of more than 10-fold from the transcriptome libraries (Table 3) were selected to verify and further analyze the gene expression changes via a series of quantitative real-time PCR (qRT-PCR) assays. The expression profiles of the selected genes were validated on oil palm seedlings in the nursery and mature palms in the field. All the total RNAs extracted from healthy and infected oil palms were free from contaminants, with intact 28S and 18S rRNAs bands (Figure 2).

**Figure 3:**
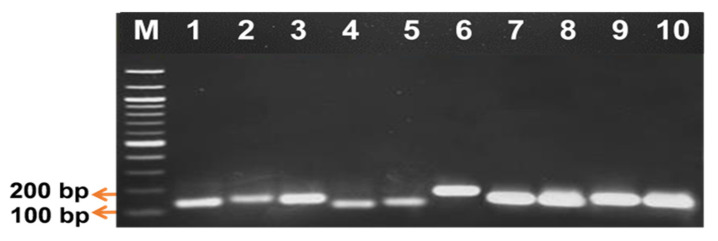
PCR amplification of oil palm root samples analyzed on 1.5% (*w*/*v*) agarose gel electrophoresis. M: 100 bp GeneRuler Marker (Fermentas, Foster City, CA, USA), Lane 1: THAUMATIN, Lane 2: NADPH, Lane 3: CHALCONE, Lane 4: MANNOSE, Lane 5: GLUCAN, Lane 6: LEUCO, Lane 7: SENESCENSE, Lane 8: FLAVONOID, Lane 9: ETYLENE, Lane 10: ANTHO.

The specificity of the 10 primer pairs was proven as they managed to amplify intact single band products within the expected amplicon length (Figure 3). The amplification specificity of each primer pair was further verified via sequencing analysis. The results showed that all the primers amplified the desired target region sequence with a high percentage of identity to the oil palm genome, ranging from 79 to 100% for most DEGs. In comparison, the other three genes had similarities from 83 to 100% with other plants (Table 4).

The efficiency of the primer pairs in amplifying specific target regions was further confirmed with the amplification efficiency test. The amplification efficiency of all the primer pairs via qPCR displayed desired efficiency values that complied with MIQE guideline [30], where the percentage of the amplification efficiency ranged from 94.6 to 109.7%, with a good correlation coefficient (R^2^ values were between 0.98 and 0.99) and the slope value ranged between −3.458 and −3.109. Moreover, the melt curve analysis produced a single peak for all the primer sets, confirming the primers’ specificity.

**Figure 4:**
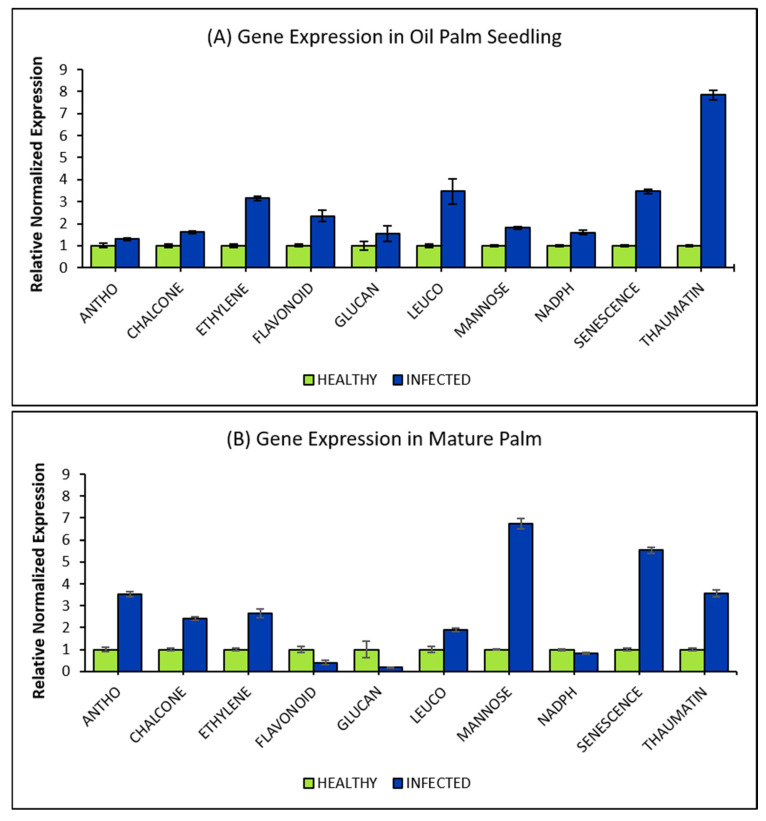
Mean of normalized expression fold for root target genes between *G. boninense*-infected tissues compared to healthy tissues. (**A**) Expression profile of root genes in nursery samples after normalization with *GRAS* and *ACTIN* reference genes and (**B**) expression profile of root genes in plantation samples after normalization with *GAPDH* and *GvHK* reference genes. The relative expression fold between two conditions being compared for both root samples was calculated by Bio-Rad C.F.X. Manager V3.0 software according to Vandesompele et al. [31].

The expression level of the selected DEGs was determined by measuring the changes in the expression of the genes using qPCR. An expression ratio of two-fold was defined as the cut-off point to reduce the high false discovery rate and false-positive results [19]. Therefore, an expression ratio higher than the cut-off value was considered as differentially expressed. The selected DEGs’ expression levels were first analyzed in 6-month-old oil palm seedlings and 15-year-old mature palms infected with *G. boninense* (Figure 4). The qPCR results of oil palm seedlings showed that the expression levels of all 10 DEGs were upregulated, ranging from 1.30-fold to 7.68-fold increased during *G. boninense* colonization as compared to the controls (Figure 4A). Among them, five of the DEGs’ abundance had increased more than 2-fold during the colonization. They were *ETHYLENE* (3.15-fold), *FLAVONOID* (2.35-fold), *LEUCO* (3.47-fold), *SENESCENCE* (3.47-fold) and *THAUMATIN* (7.68-fold). The expression profiles of these genes were further studied in oil palm roots taken from *G. boninense*-infected oil palm plantations (Figure 4B). An almost similar observation was recorded where seven DEGs (*ANTHO*, *CHALCONE*, *ETHYLENE*, *LEUCO*, *MANNOSE*, *SENESCENCE* and *THAUMATIN*) were found to be upregulated between 1.89-fold and 6.74-fold. However, the remaining three DEGs (*FLAVONOID*, *NADPH* and *GLUCAN*) appeared downregulated in the infected palms. Among the seven upregulated target genes, six of the genes, namely *ANTHO* (3.61-fold), *CHALCONE* (2.40-fold), *ETHYLENE* (2.64-fold), *MANNOSE* (6.74-fold), *SENESCENCE* (5.54-fold) and *THAUMATIN* (3.55-fold), had increased in transcript abundance more than two-fold during the fungus invasion. 

**Figure 5:**
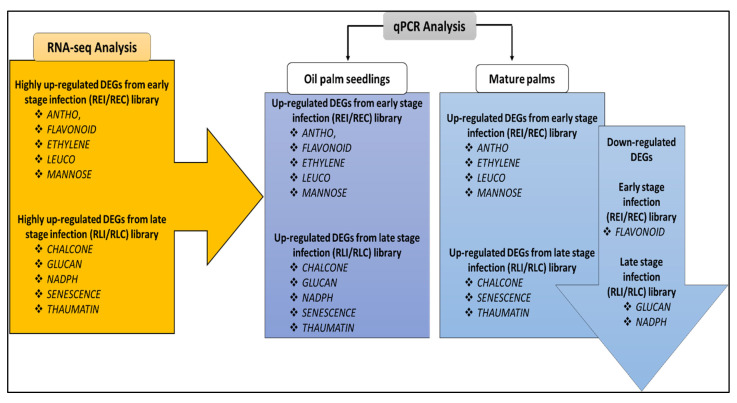
Summary of all 10 DEGs’ expression patterns obtained from RNA-seq transcriptomic libraries and their expression pattern after qPCR verification onto oil palm seedling and mature palms.

The expression pattern of all 10 DEGs on oil palm seedlings and mature palms from qPCR assay was then compared with the genes’ expression pattern obtained from RNA-seq transcriptomic libraries to confirm the obtained results from RNA-seq data. From the result, seven of the DEGs (*ANTHO*, *CHALCONE*, *ETHYLENE*, *LEUCO*, *MANNOSE*, *SENESCENCE* and *THAUMATIN*) were observed and proven to have consistent expression patterns among these two analyses, as shown in Figure 5.

## 3. Discussion

Studies on plant–pathogen interactions are crucial for the early detection of the potential threats of BSR and prolonging the economic lives of the infected trees, especially on young palms. In recent years, the RNA-seq technique has been explored to analyze the host–fungal interaction with oil palm as part of the intervention strategies for plant protection. However, critical genes involved in the network contributing to the complex genetic traits of the oil palm defense mechanism against *G. boninense* are still minimal. 

The transcriptomic libraries of *G. boninense*-infected oil palm root tissues (early-stage and late-stage infections) were successfully constructed, with a total of 126 DEGs obtained. A previous study reported that different phases of *G. boninense* infection trigger different oil palm defense-related genes to combat the BSR disease. The expression level of the genes differs throughout the infection phases [13]. Similarly, our result showed that the list and the expression level of the DEGs obtained from early infection differed from the DEGs acquired from late infection. Moreover, most of the identified DEGs were upregulated, with a more significant number of transcripts than those of downregulated genes during the infection of *G. boninense*. The amount of these transcripts increased from the early to the late infection stage. This observation may be putatively due to the escalating additional plant defense genes necessary for combating fungus infection severity during a prolonged infection course [32]. A study by Bahari et al. [13] reported that although oil palm defense mechanisms managed to weaken *G. boninense*’s thick hyphae multilayers at the early stage of infection, the plant eventually succumbed to the disease when the fungus switched to a more aggressive mode of attack via the exposure of basidiomata at the chronic infection stage. 

The putative roles and the involvement of these DEGs toward plant defense mechanisms against pathogen infection were highlighted via Gene Ontology (GO). Based on the GO term characterization, at the early stage of infection, response to nitrate and chitin catabolic processes, cytoplasmic and iron ion binding and chitinase activity were the most prominent among biological processes, cellular components and molecular functions. Meanwhile, during the late infection, the oxidation–reduction process, extracellular region and oxidoreductase were the most frequent among biological processes, cellular components and molecular functions, respectively. Our results are similar to those reported by Ho et al. [20], in which iron ion binding oxidoreductase activity, electron transport chain, vacuole and integral to plasma membrane were among the DEGs enriched during *Ganoderma* infection in oil palm. 

The integrated Kyoto Encyclopedia of Genes and Genomes (KEGG) pathway regulation network was constructed further to explore the relationship between the DEGs and the disease. The analysis using the KEGG database implied that the DEGs were involved in several pathways. Nevertheless, one common feature was plant–pathogen interaction, where the DEGs were enriched in nitrogen metabolism and xenobiotics biosynthesis pathways at early infection and late infection stages, respectively. Nitrogen, a growth-limiting nutrient for plants, is compulsory for producing all plant defense materials, either as a constituent of biosynthetic enzymes or as proteinase inhibitors, alkaloids, chitinases and glucosinolates [33]. Meanwhile, xenobiotic biosynthesis catalyzed by cytochrome P450 will be deposited in the plant vacuole or cell wall to become a physical barrier for plant defense against the pathogen [34]. 

Ten DEGs with upregulation of more than 10-fold mined from the transcriptome libraries were selected for verification via qRT-PCR assay. In the artificial inoculation experiment, the RNA of the root tissues from three biological replicates was pooled to increase the capacity to detect differential gene expression between each category of sample treatments. However, the pooled samples may not represent the population variations in the gene expression levels as this would decrease the ability to estimate within-population variation; hence, this increases both the pooling bias and false discovery rates, leading to the identification of DEGs with high false positivity rates and low positive predictive values. Due to these factors, the DEGs needed to be validated in order to confirm that the DEGs obtained from RNA-seq analysis were true positive.

Their expression levels were analyzed in 6-month-old oil palm seedlings and 15-year-old mature palms infected with *G. boninense* to observe the consistency of the DEGs’ expression patterns in the natural environment. Based on the qRT-PCR assay, there was a difference in the expression levels of the selected DEGs in mature palms (*G. boninense*-infected palms) and seedlings. The 10 DEGs were upregulated in the seedlings. Nevertheless, only seven DEGs (ANTHO, CHALCONE, ETHYLENE, LEUCO, MANNOSE, SENESCENCE and THAUMATIN) were upregulated. In contrast, the other three DEGs (*FLAVONOID*, *NADPH* and *GLUCAN*) were found to be downregulated in the infected mature palms. 

These seven DEGs, namely *ANTHO*, *CHALCONE*, *ETHYLENE*, *LEUCO*, *MANNOSE*, *SENESCENCE* and *THAUMATIN* genes, showed consistency in the transcript upregulation pattern in both seedlings and infected mature palm samples, suggesting that these DEGs were induced as a form of systemic acquired resistance (SAR). SAR is a state of preparedness that provides high resistance against pathogen attack and will become a part of plant memory to effectively protect the whole plant, especially during subsequent infections [35,36,37]. However, at this point, this is merely speculative as more studies are needed to relate the SAR nature to these DEGs. In this study, the increase in *MANNOSE* transcript abundance in seedlings and mature palm samples was expected. Mannose-binding lectins (*MANNOSE*), part of the lectin family, which is crucial for plant defense signaling during pathogen attack, was found to induce the accumulation of salicylic acid in pepper (*Capsicum annuum*) during *Xanthomonas campestris* pv vesicatoria (Xcv) infection, as well as enhancing the resistance of Arabidopsis towards *Pseudomonas syringae* pv tomato and *Alternaria brassicicola* infections [38]. Moreover, the transcript abundance of *SENESCENCE* and *THAUMATIN* were observed to have increased more significantly than the threshold value of two-fold in both *G. boninense*-infected seedlings and mature palms. The increment was anticipated as *SENESCENCE* have been shown to trigger a hypersensitive response, leading to the involvement of programmed cell death in pathogen-infected *Arabidopsis thaliana* and tobacco [39,40]. A similar observation was also reported for *THAUMATIN*, a pathogenesis-related-5 (PR-5) protein family with the ability to reduce activities in fungal cell walls related to endo-b-1,3-glucanase activity [41,42]. *THAUMATIN* has a consistent upregulation pattern with those reported in poplar [43] and *P. umbellatus* during pathogen attacks [44]. 

Interestingly, the present study revealed the upregulation of the remaining four DEGs in both oil palm samples to have robust association and involvement with the flavonoid biosynthesis pathway. Chalcone synthase (*CHALCONE*), the first key enzyme of flavonoid biosynthesis in plants involved in plant defense [45], was observed to have a parallel upregulated expression pattern with *ETHYLENE* and *ANTHO*. Ethylene, a gaseous plant hormone, has been reported to induce several plant defense response genes involved in flavonoid biosynthesis, including *CHALCONE* [46,47]. Moreover, the proportional upregulation pattern of *CHALCONE* with *ANTHO* was consistent with those reported in *Freesia hybrida* and transgenic flax, due to the significant correlation of *CHALCONE* accumulation patterns with anthocyanin, which are produced by anthocyanidin synthase (*ANTHO*) [45,48,49,50]. Additionally, the expression of *ANTHO* was observed to be inversely proportional to *LEUCO* in both seedlings and mature palms. This result is similar to the expression of these genes in transgenic poplar, where leucoanthocyanidin reductase (*LEUCO*) plays a role in producing anthocyanidin, leading to the inhibition of one another [51,52]. This explains the reason for the increment in the *ANTHO* expression ratio for nursery samples that did not achieve the cut-off value. *LEUCO* transcript abundance was highly expressed in the seedling samples and vice versa in the mature palm sample. 

In higher plants, flavonoids were categorized into six major subgroups: anthocyanins, chalcones, proanthocyanins, flavan-3-ols (catechins), flavones and flavonols [53,54]. The antioxidant activity of flavonoids is mainly determined by the structure of the flavonoid B ring [55]. Our study reported the expression pattern of flavonoid 3′-hydroxylase (*FLAVONOID*) to be upregulated in seedling samples, which is expected, as *FLAVONOID* was reported to show significant involvement in maize resistance against biotic stress [56]. However, when *FLAVONOID* transcript abundance was analyzed in infected mature palms, its expression level was shown to be downregulated. The previous report has demonstrated that *FLAVONOID* was also implicated by other stresses in adapting to external environmental conditions in Antarctic moss [57]. 

The oxidation of flavonoid moiety-like cytochrome P450 proteins (e.g., *FLAVONOID*) in producing the colored anthocyanins and stability of flavonoid compounds [58] catalyzes the NADPH- or NADH-dependent oxygenation reactions. This would influence the level of NADPH and quinone oxidoreductase-like (*NADPH*) [59]. Thus, this could explain the same direction of expression patterns between *FLAVONOID* and *NADPH* observed in both oil palm root samples. Our results also showed that the transcript abundance of *GLUCAN* has a similar expression pattern to *FLAVONOID* and *NADPH* for both samples. *GLUCAN* has been associated with diverse plant physiological and developmental processes, especially in protecting wheat cultivars from *Septoria tritici* cleaving the b-1,3-glucan in the pathogen cell wall [60]. However, since the transcript abundance of *GLUCAN* contradicts the result obtained from the previous study, this outcome could be concluded as one of the plant strategies in regulating defense mechanisms. By downregulating the production of other genes, the plant resources can be conserved and allocated to produce defense-related genes [61].

*Ganoderma* infection begins with colonizing the fungus onto the host plant cells for nutrient uptake, resulting in cell wall degradation. This cell wall penetration process continues until it becomes lethal to host cells [13,62]. Tan et al. [19] reported that when oil palm seedlings were infected with *G. boninense* from 3 to 12 weeks post-infection (wpi), several genes, such as Bowman–Birk serine protease inhibitors and dehydrin, were significantly expressed. Another study indicated the upregulation of *EgPR-1*, *EgBGIA*, *EgLYK3* and *EgEXPB18* at 3 and 7 days after inoculation but they were decreased 11 days later [13]. These observations on *G. boninense*’s interaction with oil palm revealed that a diverse set of defense-related genes were differently expressed at different time points of fungal infection. Moreover, it is stipulated that oil palm most likely overcome BSR infection in the early stage via the upregulation of several multifaceted defense-related genes but are later overwhelmed by the aggressive changes in the fungus mode of infection at the later stage of colonization (usually observed via the appearance of disease symptoms such as the emergence of chronic-stage fruiting bodies); thus, a new set of defense-related genes were induced as the host’s final counter-measure against this pathogen. 

These results indirectly highlighted the importance of identifying defense-related genes associated with the onset of early internal symptoms and late symptoms in oil palm. In the present study, seven genes were found to be differentially expressed in *G. boninense*-infected seedlings and mature palms, consistent with the expression patterns of the transcriptomic libraries. These indicate that the gene products of *ANTHO*, *LEUCO*, *ETHYLENE* and *MANNOSE* may be involved in the defense mechanism of oil palm during the initial stage of *G. boninense* infection upon the formation of lesions on the plant roots. In contrast, *CHALCONE*, *SENESCENCE* and *THAUMATIN* may be involved during a later stage of *G. boninense* infection. Therefore, these seven genes may have direct involvement in the oil palm defense mechanism against *G. boninense*.

## 4. Materials and Methods

### 4.1. Plant Materials and Treatments for Transcriptomic Profiling

The root tissues used for the transcriptome library generation were sampled from 12-month-old commercial oil palm seedlings, *Tenera* (DxP), which were artificially infected with *Ganoderma boninense* colonized on rubber woodblock (RWB) using a root inoculation technique [63]. The RWBs were inoculated with a 7-day-old pure culture of *Ganoderma boninense* PER71. *G. boninense* strain PER71 is an aggressive fungal pathogen that causes BSR. The fungus was first isolated from an infected oil palm in a plantation in Perak [63]. Briefly, the autoclave-sterilized RWBs with dimensions of 3 × 3 × 6 cm were submerged in 50 mL liquid malt extract agar (MEA) and left to solidify overnight. Then, the RWBs were inoculated with a 7-day-old pure culture of *Ganoderma boninense* PER71. The inoculated RWBs were incubated at 28 °C in the dark for 3 months to allow complete colonization of the *G. boninense* mycelia towards the RWBs. Successful colonization of *G. boninense* was determined by visual evidence of complete mycelial covering the RWBs. These colonized RWBs were inoculated to oil palm seedlings by firmly placing the oil palm root bulb in contact with standing RWB inocula that were covered with soil in a separated polybag. The RWBs were placed in direct contact with oil palm roots in plastic bags to avoid root contact with external inoculum sources. A negative control was prepared by inoculating the oil palm seedlings with uninoculated RWBs. The oil palm seedlings were subjected to two different treatments: oil palm treated with RWBs fully colonized with *G. boninense* and oil palm treated with empty RWBs (uninfected block; as controls), with three biological replicates for each treatment. Based on the disease severity index (DSI) on a scale of 0 (healthy-looking palm without any fungal mycelium appearance) to 4 (dead palm) [64,65], root samples of each treatment were harvested at two different scales: scale of 1 (observation of lesions and mycelia on the root surface at 3 to 12 weeks post-inoculation (wpi) without any foliar symptoms) and scale of 3 (disease symptom observation, such as yellowing of leaves and formation of fruiting bodies on the stem). The samples were labeled as either early infection (scale 1) or late infection (scale 3).

### 4.2. Total RNA Extraction and cDNA Synthesis

Total RNA from the roots was extracted using the cetyl trimethylammonium bromide (CTAB) protocol [66,67]. The RNA was treated with DNase 1 from the RQ1 RNase-Free DNase Kit (Promega, Madison, WI, USA). The concentration and the quality of the total RNAs were determined using NanoDrop^®^ ND-1000 Analysis (Thermo Scientific, Wilmington, DE, USA). The integrity of the RNA was determined via the Agilent 2100 Bioanalyzer (Agilent Technologies, Santa Clara, CA, USA). First-strand cDNA was synthesized using the GoScript™ Reverse Transcription Kit (Promega, Madison, WI, USA) according to the manufacturer’s protocol.

### 4.3. RNA-Sequencing and Transcriptome Library Generation

The RNAs with a high RNA integrity number (RIN) were subjected to RNA sequencing using the Illumina platform (Hiseq 2000). The paired-end raw data from the RNA-seq were converted to FASTAQ format. The reads were later trimmed based on the Phred 20 quality score (Q score) and the sequence length of a minimum 50 bp via the DynamicTrim and LengthSort software. The reads in control and treated samples were then screened for the phiX reads (phiX spike) and other contaminants, such as vector sequences, using Bowtie2 software [68]. The filtered raw data reads were mapped against the oil palm reference genome using the TopHat, Bowtie and Cufflinks packages [69]. The merged assemblies were fed to Cuffdiff and the expression comparisons were calculated and filtered according to the parameters of log_2_ fold change (FC) ratio of the infected to non-infected root samples, with adjusted *p*-values of 0.05 (*p* < 0.05) and false discovery rate (FDR) < 0.05 [20]. The DEG mode of expression (highly or lowly expressed) of the samples being compared were determined via the fragments per kilobase of transcript per million reads mapped (FPKM) method [29]. The genes with a positive log_2_FC value greater than 2.0 with a *p*-value < 0.05 were defined as significantly upregulated DEGs, whereas DEGs with negative log_2_FC values smaller than −2.0 indicated that the genes were downregulated in the two samples being compared. The candidate genes then were mapped to the Gene Ontology (GO) and Kyoto Encyclopedia of Genes and Genomes (KEGG) databases with Blast2GO (B2G) (http://www.blast2go.com/; accessed on 1 April 2017) [70,71]. The RNA-seq data generated from the study are curated in a publicly accessible database, the Genomsawit website (http://genomsawit.mpob.gov.my/ accessed on 1 December 2020), an official portal of the Malaysian Oil Palm Genome Programme (MyOPGP).

### 4.4. Quantitative Real-Time Polymerase Chain Reaction (qRT-PCR) Analysis

Ten oil palm DEGs with more than 10-fold upregulation, identified from the libraries, were selected for further expression verification analysis via qRT-PCR on root samples of 6-month-old oil palm seedlings and 15-year-old oil palms infected with BSR. The root samples were harvested from palms of the disease-free nursery (control) and palms from *G. boninense*-infected plantations. 

The samples were taken based on two different conditions: healthy samples (Uninfected/Control) and samples infected with *G. boninense* (Infected/Treated). The infected samples were selected according to visual external symptoms such as the wilting of leaves as well as the formation of fruiting bodies on the oil palm stem. For nursery samples, both the healthy and infected tissues were sampled from 6-month-old seedlings grown at MPOB-UKM nursery, Bangi, Selangor. As for the field samples, the primary root tissues were sampled from 15-year-old oil palms at two different locations: the healthy tissues were taken from MPOB Keratong oil palm plantation, FELDA Keratong, Pahang, while the infected tissues were taken from FELCRA oil palm plantation, Seberang Perak, Perak.

The specific primers of the DEGs were designed using Primer Premier 5.0 software (Premier Biosoft International, San Francisco, CA, USA), as listed in Table 5.

The PCR was carried out in 20 µl reaction mixture using the *Taq* DNA polymerase kit (Fermentas, Foster City, CA, USA) according to the manufacturer’s protocol and run in a thermal cycler according to the following programs: 2 min initial denaturation at 95 °C; followed by 35 cycles of 1 min at 94 °C, 30 s at 61 °C, 1 min at 72 °C; a 5 min final extension at 72 °C and held at 15 °C. The amplicons were electrophoresed in 1.5% agarose gel and visualized under UV light transillumination using a BioImaging System (UVP, Upland, CA, USA). 

Using nuclease-free water, the cDNAs were subjected to 4-fold serial dilutions (100 ng, 25 ng, 6.25 ng, 1.56 ng and 0.39 ng). The qPCR was carried out using the SensiFast™ SYBR^®^ No-ROX One-Step Master Mix Kit (2X) (Bioline, Memphis, TN, USA) according to the manufacturer’s protocol and performed in triplicate. The test was carried out using the Bio-Rad CFX96 Real-time PCR thermocycler (Bio-Rad, Hercules, CA, USA) with the cycling conditions as follows: 95 °C of initial denaturation for 2 min, 40 cycles of 95 °C for 5 s and 61 °C for 30 s followed by dissociation curve programmed at 65 °C at 5 s and 95 °C for 5 s with a heating rate increment of 0.1 °C per 1 s. Two negative controls were included: no reverse transcriptase (NRT) and non-template control (NTC). The result of the primer efficiency was analyzed using Bio-Rad C.F.X. Manager V3.0 software and the PCR amplification efficiency (E) was calculated based on the slope data of the primer pair’s standard curve of a semi-log regression line plot of Cq value vs. log of input cDNA with the following formula (Equation (1)) [72]:

E = [10^(−1/slope)^ − 1] × 100% (1)

A yield of a 10-fold increase in PCR products every 3.32 cycles during the exponential phase of amplification was considered 100% efficient [73]. 

The expression profile of the target genes was determined using the Bio-Rad CFX96 Real-time PCR thermocycler (Bio-Rad, Hercules, CA, USA). Each PCR (20 μL; in quadruplicate) contained 7.0 μL of nuclease-free water, 0.4 μM of each forward and reverse gene-specific primer, 1× SensiFast™ SYBR^®^ No-ROX Master Mix Kit and 1 μL of cDNA and was run in the thermocycler with the cycling conditions as follows: 95 °C of initial denaturation for 2 min, 40 cycles of 95 °C for 5 s and 61 °C for 30 s. The graph of expression folds of the DEGs in the treated samples was constructed according to Cq values using the Bio-Rad C.F.X. Manager V3.0. The cDNA samples were diluted and normalized with the endogenous reference genes, *GRASS, GAPDH*, *GvHK* and *ACTIN* genes [67]. The relative transcript levels were calculated based on geNorm statistical algorithms [31]. Samples that produced a higher cycle threshold (CT) value relative to the control were downregulated and samples that produced a lower CT value relative to the control were considered to be upregulated. The calculated CT values were the means of triplicate measurements. The comparative fold differences (CT quantification) were calculated using the ΔΔ𝐶𝑇 method [74,75].

## 5. Conclusions

RNA-seq analysis has been used to successfully identify DEGs related to the oil palm defense mechanism in response to *G. boninense* infection. The DEGs selected from the transcriptomic libraries were proven to produce a consistent upregulation pattern across independent samples of infected seedlings and mature palms when validated using qRT-PCR. To the best of our knowledge, this is the first study to report the validation of DEGs mined from *G. boninense*-infected oil palms’ transcriptomic libraries across independent biological replicates subjected to RNA-seq. However, the expression patterns of these genes need to be further elucidated on a larger sample size including different BSR infection stages taken from various field sites.

## Figures and Tables

**Table 1:** Total raw paired-reads of RNA-seq and the filtered reads after the quality assessments and trimming and removal of PhiX contaminants for oil palm root tissues under different treatments.

Samples	Total Reads (bp)	Low-Quality Reads (%)	Total High-Quality Reads (bp)	PhiX Contamination (%)	Total Sample Reads (bp)
REI	16,536,956	8.51	15,129,373	0.32	15,080,896
RLI	21,075,290	9.49	19,075,010	0.31	19,014,726
REC	17,741,550	8.71	16,195,459	0.33	16,141,333
RLC	27,247,240	10.79	24,305,602	0.32	24,227,629

REI = root early infection; RLI = root late infection; REC = root early control; RLC = root late control.

**Table 2:** Summary of total significant differentially expressed genes (DEGs) for oil palm root samples pair and the number of transcripts expressed.

Oil Palm Root Sample Pairs	Total Significant DEGs	Up-Regulated Transcripts	Down-Regulated Transcripts
REI/REC	21	12	9
RLI/RLC	103	69	34

REI/REC = root early infection/root early control; RLI/RLC = root late infection/root late control.

**Table 3:** List of the selected differentially expressed genes in oil palm root samples at different stages of *G. boninense* infection.

Gene ID	Annotation Genes	Labels	FPKM		Log 2 Fold-Change
			Control	Treated	
**Highly Upregulated Root Genes at Early Stage of Infection (REI/REC)**					
XLOC_022484	anthocyanidin synthase	*ANTHO*	5.95	189.31	31.79
XLOC_009804	leucoanthocyanidin reductase-like	*LEUCO*	16.66	198.57	11.91
XLOC_019742	ethylene-responsive transcription factor 1b-like	*ETHYLENE*	4.09	109.77	26.85
XLOC_005112	flavonoid 3–hydroxylase	*FLAVONOID*	0	19.56	Positive Infinity
XLOC_013737	mannose-specific lectin-like	*MANNOSE*	85.08	1114.43	13.09
**Highly Upregulated Root Genes at Late Stage of Infection (RLI/RLC)**					
XLOC_016957	chalcone synthase	*CHALCONE*	0	674.91	Positive Infinity
XLOC_009482	glucan endo-beta-glucosidase-like	*GLUCAN*	555.34	9543.37	17.18
XLOC_009990	nadph:quinone oxidoreductase-like	*NADPH*	5.69	107.89	18.96
XLOC_017303	senescence-associated partial	*SENESCENCE*	91.86	1540.91	16.77
XLOC_001542	thaumatin-like protein	*THAUMATIN*	13.10	187.77	14.33

**Table 4:** BLASTX of genes of interest for oil palm root against the oil palm genome database.

Gene	Amplicon Size (bp)	BLAST Results	Score (Bits)	E-Value	Percentage of Identity (%)	Accession No.
*ANTHO*	138	Anthocyanidin synthase (*Delphinium grandiflorum*)	51.6	9 × 10^−7^	92	BAO04186.1
*LEUCO*	170	PREDICTED: leucoanthocyanidin reductase-like(*Elaeis guineensis*)	72.4	4 × 10^−14^	100	XP 010916146.1
*ETHYLENE*	142	PREDICTED: ethylene-responsive transcription factor 1B-like (*Elaeis guineensis*)	70.5	2 × 10^−13^	100	XP 010904582.1
*FLAVONOID*	155	flavonoid 3′–hydroxylase (*Sorghum bicolor*)	56.2	3 × 10^−8^	83	ABG54321.1
*MANNOSE*	105	PREDICTED: mannose-specific lectin-like(*Elaeis guineensis*)	43.9	3 × 10^−4^	100	XP 010910930.1
*CHALCONE*	135	PREDICTED: chalcone synthase-like (*Elaeis guineensis*)	48.1	9 × 10^−5^	85	XP 010910945.1
*GLUCAN*	110	PREDICTED: glucan endo- 1,3-beta-glucosidase-like *(Elaeis guineensis*)	36.2	0.67	79	XP 010921890.1
*NADPH*	116	PREDICTED: NADPH:quinone oxidoreductase-like *(Elaeis guineensis*)	40.8	0.010	100	XP 010922202.1
*SENESCENCE*	169	putative senescence-associated protein (*Cupressus sempervirens*)	90.5	1 × 10^−21^	100	ACA30301.1
*THAUMATIN*	110	PREDICTED: *Elaeis guineensis* thaumatin-like protein (LOC105033331), mRNA	75.0	2 × 10^−10^	94	XM 010908090.1

ANTHO = Anthocyanidin synthase; CHALCONE = Chalcone synthase; ETHYLENE = Ethylene-responsive transcription factor 1b-like; FLAVONOID = Flavonoid 3–hydroxylase; GLUCAN = Glucan endo-beta-glucosidase-like; LEUCO = Leucoanthocyanidin reductase-like; MANNOSE = Mannose-specific lectin-like; NADPH = NADPH: quinone oxidoreductase-like; SENESCENCE = Senescence-associated partial; THAUMATIN = Thaumatin-like protein.

**Table 5:** List of primer sequences of the selected DEGs at different stages of *G. boninense* infection.

Primer Name	Primer Sequence (5′–3′)		Product Length (bp)
	Forward Primer	Reverse Primer	
*ANTHO*	ACAACATGGTCCCCGGTCT	GGTGGAGGATGCTCTTGTAGGT	138
*LEUCO*	TCCGTTTTGGGCGGTTCT	CGGCGGACTTTCCTCTTTTC	170
*ETHYLENE*	AAGAGCAAGGCAGGGAATGG	CTTCTGCGCTGTCAAAGGTTC	142
*FLAVONOID*	GTTTGTGGTGGGAGACTTCGTG	CCTCATTCTGCTCGGTTGGAC	155
*MANNOSE*	TCGGATGGGAACCTTGTGG	CCGATCTCGTTGGAGGATACAG	105
*CHALCONE*	GAGCAGATCCAATGCAAGGTGT	GGTTGAGGAGGTGGAAGGTGA	135
*GLUCAN*	AGCAAGCTACTGGGTCCAAAC	GCACATACTGGGCTTTATCTCC	110
*NADPH*	CGAGATTGATGGCAAGTGTCC	TCAGAGGAGCTGGGATGGAGT	116
*SENESCENCE*	GGCACGGCCATCAGTAGAGTA	AGCCAAGCGTTCATAGCGAC	169
*THAUMATIN*	ACGAGGGAGATGTCGATGAA	GACTGCGGTGGTAAACTTGC	110

## Data Availability

The data is contained within this article.
